# Investigation of the Robustness of Rayleigh Optical Activity for the Assignment of Absolute Configurations of Chiral Molecules

**DOI:** 10.1021/acs.jpca.5c08390

**Published:** 2026-02-25

**Authors:** Andrew R. Puente, Duncan McArthur, Emmanouil I. Alexakis, Lewis E. MacKenzie, Robert P. Cameron, Laurence D. Barron, Prasad L. Polavarapu

**Affiliations:** 1 Department of Chemistry, 5718 Vanderbilt University, Nashville, Tennessee 37235, United States; 2 SUPA and Department of Physics, 3527 University of Strathclyde, Glasgow G4 0NG, U.K.; 3 Department of Pure and Applied Chemistry, 3527 University of Strathclyde, Glasgow G1 1RD, U.K.; 4 Department of Chemistry, 3526 University of Glasgow, Glasgow G12 8QQ, U.K.

## Abstract

Experimental measurements of Rayleigh optical activity (RayOA) for liquid phase chiral molecules have been recently reported for the first time, nearly 50 years after it was theoretically formulated. Inspired by these experimental data, we computationally model the RayOA of several chiral molecules to assess the usefulness of this newly reported experimental method for assigning their absolute configurations. We consider the influence of factors that can often preclude the routine assignment of absolute configurations, including conformational flexibility, solute–solvent clusters, and dispersion interactions. We find that RayOA is not as sensitive to these factors as other commonly used chiroptical spectroscopies, namely, specific rotation, electronic circular dichroism, vibrational circular dichroism, and vibrational Raman optical activity, which suggests that RayOA may be best suited for routine absolute configuration assignment. Additionally, we report on a class of chiral propellers that could be tailored to have large magnitudes of RayOA. We also find that there can be preresonance RayOA enhancement when the wavelength of RayOA measurement approaches that of electronic transitions. Thus, RayOA may potentially find a strong foothold within the chemical community due to its potential robustness and ease of computational treatment for absolute configuration determinations.

## Graphical abstract

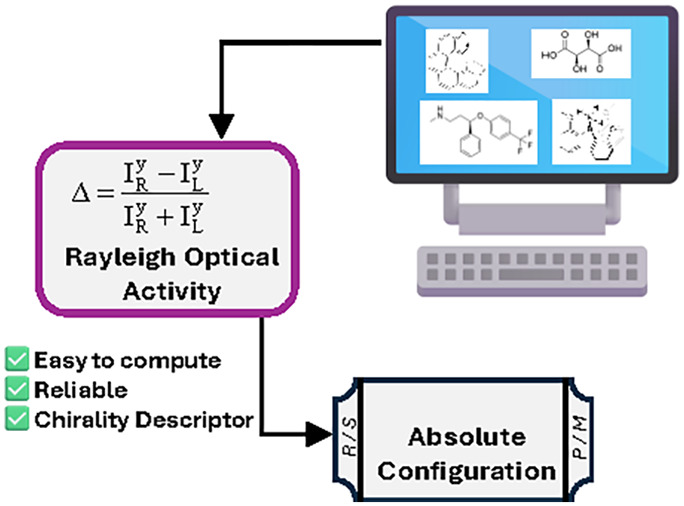

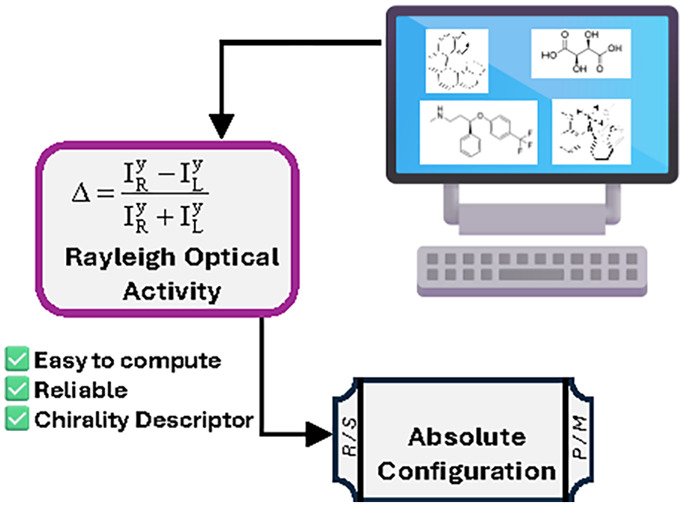

## Introduction

Four different chiroptical techniques are currently used to assign the absolute configuration of chiral molecules.^1,2^ These are specific optical rotation, commonly referred to as specific rotation (SR),^3^ electronic circular dichroism (ECD),^4^ vibrational circular dichroism (VCD),^5–7^ and vibrational Raman optical activity (ROA).^8,9^ For chiral compounds in the solution phase, SR is derived from experimentally measured optical rotation (OR) by dividing it with concentration (in g/cm^3^) and path length (in dm). For practical applications, SR is used by many synthetic chemists on an empirical basis and more recently by physical chemists using quantum mechanical calculations for assigning the absolute configuration of chiral molecules. The difficulties in this endeavor arise from different directions. (a) Experimental observation of the sign of SR can depend on the solvent and concentrations employed; (b) SR is directly proportional to the trace of the electric-dipole–magnetic-dipole–polarizability tensor.^8^ Thus, accurate predictions of SR rely on the near cancellation of the diagonal elements of this tensor. As a result, any perturbations that slightly change these diagonal elements can lead to an inversion of SR. Additional issues arise when attempting to compute small SR values, as predictions can be off by an order of magnitude or worse, leading to difficulties in correctly predicting the sign of SR through computation. Because of these and other issues in obtaining accurate computations of SR, the complementary spectroscopic techniques ECD, VCD, and ROA are often used by chiroptical spectroscopists for the determination of absolute configurations. However, all four methods mentioned above require extensive conformational analysis and rigorous computational treatment of conformers to achieve an adequate agreement with experimental data. This is especially challenging when strong solute–solvent interactions are present, such as in methanol or dimethyl sulfoxide (DMSO) solvent, as solvent molecules must be incorporated into calculations and the solute–solvent conformational space must be explored, which is nontrivial. In addition, the inclusion of dispersion interactions into theoretical predictions of these chiroptical properties often leads to varying conclusions.^10^ Given these difficulties with currently used chiroptical techniques, there is a definite interest in finding new methods that are insensitive, or at least less sensitive, to conformational flexibility, solute–solvent interactions, and dispersion interactions.

Rayleigh optical activity (RayOA) represents differential Rayleigh scattering responses toward right and left circularly polarized radiation by chiral molecules. Polarized Rayleigh scattering was theoretically formulated by Atkins and Barron^11^ in 1969. Two years later, Barron and Buckingham^12^ developed the theory of circularly polarized Rayleigh scattering by chiral molecules. Soon after the theoretical formulation of RayOA, a two-group model was developed by Barron and Buckingham.^13^ A dimensionless circular intensity differential (CID), Δ, was defined as the ratio of circular intensity difference to the circular intensity sum. For incident right and left circular polarizations, the scattered light in the 90° scattering geometry with polarization parallel to the scattering plane, referred to as depolarized RayOA, the CID is labeled as Δ_*z*_ and that for the scattered light with polarization perpendicular to the scattering plane as polarized RayOA and respective CID is Δ_*x*_. The two-group model enabled predictions of sign and magnitude of CIDs expected for two groups, with 3-fold or higher rotation axes, oriented in a chiral disposition. This model was used by Barron^14^ to predict that for hexahelicene, the magnitudes are Δ_*z*_ = 6.2 × 10^–4^ and Δ_*x*_ = 4.1 × 10^–5^ and those for biphenyl twisted at 45° are, respectively, 1.3 × 10^–3^ and 6 × 10^–5^. Barron also predicted that a molecule with a large SR will not necessarily have a large RayOA CID.^14,15^ In 1985, Barron and Johnston developed the theory of rotational ROA for symmetric top molecules and showed that the CID for integrated rotational ROA equals that for Rayleigh CID.^16^ In this context, Barron and Johnston used triphenylborane with *D*_3_ symmetry as a model molecule and predicted the RayOA magnitudes of Δ_*z*_ = 2.61 × 10^–3^ and Δ_*x*_ = 1.97 × 10^–5^. There was a long quiet period in the literature on RayOA since that time. In 2008, Zuber et al. undertook quantum mechanical calculations of RayOA and suggested its use for absolute configuration determination.^17^ More recently, a theory for Rayleigh–Brillouin optical activity applicable to liquids has been developed.^18^

Although experimental measurements on differential scattering of circularly polarized light were published for various artificial and biological aerosol particles (of ∼100 μm size)^19^ and organized chiral biological macromolecular assemblies of size greater than λ/20,^20^ these measurements^21^ were not associated with the RayOA of chiral molecules. Until recently, RayOA measurements of condensed phase chiral molecules themselves were not explored. With latest advances in experimental measurement of RayOA for chiral molecules in the liquid phase,^22^ there is a need for investigating the usefulness and applicability of RayOA. To this end, we report here the computational evaluations of the sensitivity of RayOA to (a) the use of simple theoretical models, (b) conformational rigidity, (c) preresonance, (d) conformational flexibility, (e) dispersion interactions, (f) higher order symmetries, and (g) solute–solvent clusters.

Some of the calculations reported here were taken from the Ph.D. dissertation of one of the authors.^23^

## Theoretical Background

In this section, we provide the theoretical background needed for the computation of RayOA. Most of the equations given here are taken from Barron,^8^ with some modifications to the notation as used by Polavarapu.^24,25^

Light scattering arising from the interaction of chiral molecules with circularly polarized light involves the induced electric dipole moment, μ_α_, magnetic dipole moment, *m*_α_, and electric quadrupole moment, Θ_αβ_. These moments arising from electric field **F**, magnetic field **B**, and their time derivatives can be given, relevant for this work, as^8^

$$\mu_{\alpha }=\mu_{\alpha }^{0}+\alpha_{\alpha \beta }F_{\beta }+\omega^{-1}G_{\alpha \beta }^{\prime }{Ḃ}_{\beta }+...$$ 1

$$m_{\alpha }=m_{\alpha }^{0}-\omega^{-1}G_{\beta \alpha }^{\prime }{Ḟ}_{\beta }+...$$ 2

$$\Theta_{\alpha \beta }=\Theta_{\alpha \beta }^{0}+A_{\gamma \alpha \beta }F_{\gamma }+...$$ 3

The first term on the right-hand side, in each of the above three equations, represents the respective moment in the absence of fields. In eqs 1–3, α_αβ_ is the electric dipole–electric dipole (EDED) molecular polarizability tensor, ω^–1^ *G*_αβ_^′^ is the electric dipole–magnetic dipole (EDMD) polarizability tensor, and *A*_αβγ_ is the electric dipole–electric quadrupole (EDEQ) polarizability tensor, as defined below.

The frequency-dependent EDED polarizability tensor α_αβ_ is given by eq 4:

ααβ=∑m≠n2ωmnRe{⟨ψn0|μ̂α|ψm0⟩⟨ψm0|μ̂β|ψn0⟩}ℏ(ωmn2−ω2) 4

where μ̂_α_ is the electric dipole moment operator, ψ_*n*_° is the wavefunction of the *n*th state, ω_*mn*_ is the transition frequency between states *m* and *n*, ω is the angular frequency of incident light, and ℏ = *h*/2π, in which *h* is Planck’s constant. Similarly, the frequency-dependent EDMD polarizability, ω^–1^ *G*_αβ_^′^, is given by eq 5:

ω−1Gαβ′=−∑m≠n2Im{⟨ψn0|μ̂α|ψm0⟩⟨ψm0|m̂β|ψn0⟩}ℏ(ωmn2−ω2) 5

where *m̂*_β_ is the magnetic dipole moment operator. The frequency-dependent EDEQ polarizability tensor, *A*_αβγ_, is given by eq 6:

Aαβγ=4πh∑n≠mωmnωmn2−ω2Re{⟨ψn0|μ̂α|ψm0⟩⟨ψm0|Θ̂βγ|ψn0⟩} 6

where Θ̂_βγ_ is the electric quadrupole moment operator. The SR of chiral molecules in isotropic medium is determined by the trace of the ω^–1^ *G*_αβ_^′^ tensor in eq 5.

The anisotropy of the EDED polarizability tensor is given as

$$\beta^{2}=\frac{1}{2}\left\{{\left(\alpha_{xx}-\alpha_{yy}\right)}^{2}+{\left(\alpha_{xx}-\alpha_{zz}\right)}^{2}+{\left(\alpha_{yy}-\alpha_{zz}\right)}^{2}+6\left[{\alpha_{xy}}^{2}+{\alpha_{xz}}^{2}+{\alpha_{yz}}^{2}\right]\right\}$$ 7

The anisotropy of the product of EDED polarizability and EDMD polarizability tensor elements is given as

$$\omega^{-1}\gamma^{2}=\left(\frac{\omega^{-1}}{2}\right)\left\{\left(\alpha_{xx}-\alpha_{yy}\right)\left(G_{xx}^{\prime }-G_{yy}^{\prime }\right)+\left(\alpha_{xx}-\alpha_{zz}\right)\left(G_{xx}^{\prime }-G_{zz}^{\prime }\right)+\left(\alpha_{yy}-\alpha_{zz}\right)\left(G_{yy}^{\prime }-G_{zz}^{\prime }\right)+3\left[\alpha_{xy}\left(G_{xy}^{\prime }+G_{yx}^{\prime }\right)+\alpha_{xz}\left(G_{xz}^{\prime }+G_{zx}^{\prime }\right)+\alpha_{yz}\left(G_{yz}^{\prime }+G_{zy}^{\prime }\right)\right]\right\}$$ 8

The anisotropy of the product of EDED polarizability and EDEQ polarizability tensor elements is given as

$$\omega^{-1}\delta^{2}=\frac{1}{2}\left\{\left(\alpha_{yy}-\alpha_{xx}\right)A_{zxy}+\left(\alpha_{xx}-\alpha_{zz}\right)A_{yzx}+\left(\alpha_{zz}-\alpha_{yy}\right)A_{xyz}+\alpha_{xy}\left(A_{yyz}-A_{zyy}+A_{zxx}-A_{xxz}\right)+\alpha_{xz}\left(A_{yzz}-A_{zzy}+A_{xxy}-A_{yxx}\right)+\alpha_{yz}\left(A_{zzx}-A_{xzz}+A_{xyy}-A_{yxy}\right)\right\}$$ 9

To eliminate the dependence on incident laser intensity, associated constants, and instrumental parameters, it is customary to report dimensionless circular intensity differential (CID), Δ, as the ratio of circular intensity difference, *I*_α_^γ^ – *I*_β_^δ^, to the circular intensity sum, *I*_α_^γ^ + *I*_β_^δ^:

$$\Delta =\left(\frac{I_{\alpha }^{\gamma }-I_{\beta }^{\delta }}{I_{\alpha }^{\gamma }+I_{\beta }^{\delta }}\right)$$ 10

where superscripts indicate the polarization of incident laser light and subscripts indicate that of scattered light. In eq 10, the numerator represents Rayleigh circular intensity difference activity, while the denominator represents the corresponding Rayleigh scattering activity.

The equations presented so far are applicable to RayOA. Equations 7–10 are also applicable for ROA when polarizabilities are replaced with their respective normal coordinate derivatives.

RayOA can be measured in any of the many experimental geometries that ROA has been considered. These include incident circularly polarized (ICP),^9^ scattered circular polarization (SCP),^26^ and dual circular polarization (DCP)^27^ geometries. In ICP geometry, the incident laser light is changed between right and left circular polarizations and the scattered light with a chosen linear polarization^9,28,29^ is measured and its difference taken. In SCP geometry, the incident laser light has linear polarization (or is unpolarized), and the scattered light with right and left circular polarizations is measured separately and its difference taken. In SCP geometry, one can use either 90° right angle scattering geometry or 180° back scattering geometry. In DCP geometry, the incident laser light is changed between right and left circular polarizations, and the scattered light with right and left circular polarizations is measured and its difference taken. Here, one may collect right (or left) circularly polarized scattered light when incident laser polarization is also right (or left) circularly polarized, which is referred to as DCP_I_, or their asymmetric combinations, which is referred to as DCP_II_. Appropriate expressions for CIDs for all these geometries are provided by Barron,^8^ Nafie,^7^ and Polavarapu.^24,25^

Since the current experimental RayOA measurements are done^22^ for the right-angle SCP measurement, we will describe the RayOA CID equation relevant for this geometry. For the 90° scattering geometry,

$$\frac{I_{z}^{R}-I_{z}^{L}}{I_{z}^{R}+I_{z}^{L}}=\frac{I_{R}^{y}-I_{L}^{y}}{I_{R}^{y}+I_{L}^{y}}=\frac{\frac{16}{3}\left(\frac{2\pi }{\lambda }\right)\left(3\omega^{-1}\gamma^{2}-\omega^{-1}\delta^{2}\right)}{8\beta^{2}}$$ 11

where λ is incident laser wavelength. In eq 11, subscript *z* identifies the polarization axis of scattered light and the propagation direction of incident light; superscript *y* identifies the polarization axis of incident light and also the propagation direction of scattered light, implying that the *yz*-plane is the scattering plane.

The advantage of the form of equations given here is that the α_αβ_, ω^–1^ *G*_αβ_^′^, and *A*_αβγ_ tensor elements can be taken directly from outputs of the Gaussian program, where α_αβ_ (listed as Property number 1 - Alpha­(−w,w)) is in units of bohr^3^ and ω^–1^ *G*_αβ_^′^ (listed as Property number 2 - FD Optical Rotation Tensor) and *A*_αβγ_ (listed as Property number 4 - D-Q polarizability) are both in units of Bohr^4^. Then eqs 7, 8 and 9 come in units of bohr^6^, bohr^7^, and bohr^7^, respectively, so λ in eq 11 will be in units of bohr.

## Computational Details

All optical tensors were computed by using an input wavelength of 532 nm unless otherwise indicated. This input wavelength is consistent with that being used in current experimental RayOA measurements.^22^ For wavelength-dependent studies, the following wavelengths are used: 365, 405, 436, 532, 546, 589, 633, 799, and 1064 nm. These wavelengths were chosen based on common laser wavelengths and wavelengths reported for SR measurements. All quantum chemical calculations of dynamic polarizability tensors were done using Gaussian 16 ^30^ using the options “polar = optrot CPHF = Rdfreq IOp­(10/46 = 7)”. An in-house-developed Python code was used for RayOA calculations, which reads the appropriate tensor data from Gaussian outputs. The equations needed for RayOA calculations are also coded into an Excel sheet for cross-verification of the computed RayOA CID values.

When uncertainties associated with Boltzmann population weighted SR and RayOA are presented, they are computed as standard error, σ, in the weighted average using the formula

$$\sigma^{2}=\frac{\left\langle X^{2}\right\rangle -{\left\langle X\right\rangle }^{2}}{N}$$ 12

The definitions for quantities in eq 12 are given in eqs 13 and 14:

$$\left\langle X\right\rangle =\frac{\underset{i}{\sum }X_{i}e^{-E_{i}/RT}}{\underset{i}{\sum }e^{-E_{i}/RT}}$$ 13

$$\left\langle X^{2}\right\rangle =\frac{\underset{i}{\sum }{X_{i}}^{2}e^{-E_{i}/RT}}{\underset{i}{\sum }e^{-E_{i}/RT}}$$ 14

In eqs 13 and 14, the summation runs over the number of conformer geometries used for Boltzmann weights, *i* = 1, 2, ..., *N*, and *X_i_* represents either SR or RayOA for the *i*th geometry, with energy *E_i_*. Uncertainties are presented as ±σ.

Molecular visualizations were made with CYLview20.^31^

## Results and Discussion

The molecules investigated in this work are displayed in Figure 1.

**Figure 1:**
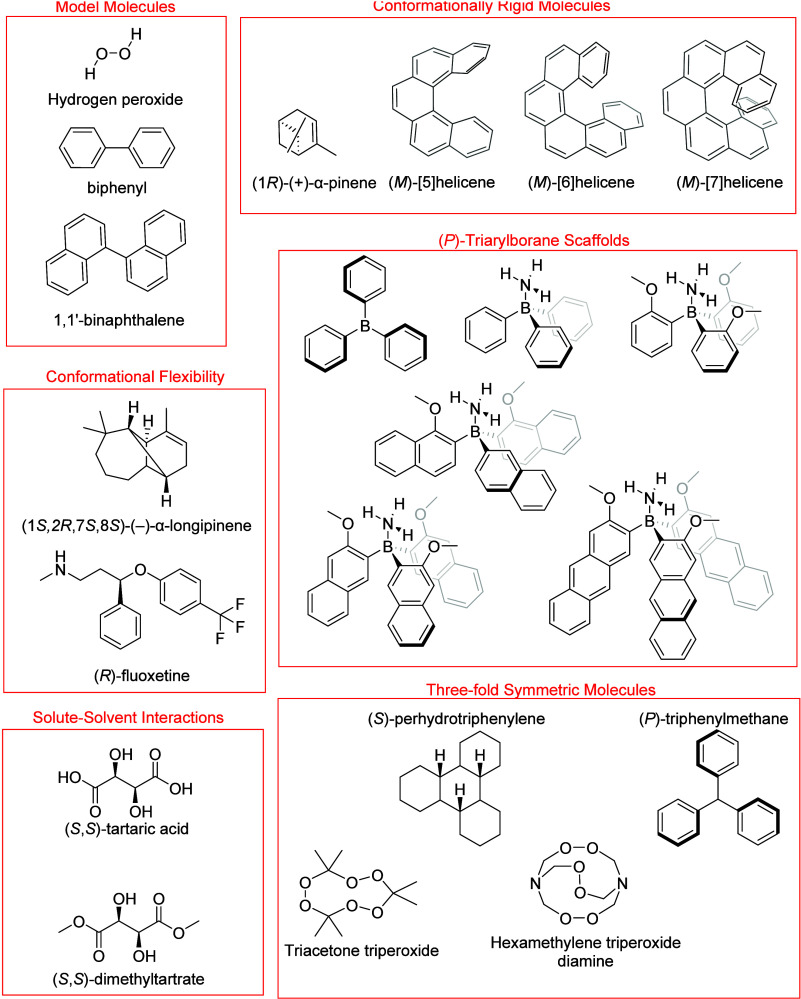
Structures of molecules in this study. Molecules are organized based on the section in which their RayOA is discussed. (*M*)-handedness is used for helicenes. Propeller handedness used for triarylboranes and triphenylmethane is right-handed. Triacetone triperoxide structures used both (*P*,*P*,*P*) and (*M*,*P*,*P*) chiralities. Hexamethylene triperoxide diamine structures used (*M*,*M*,*M*) and (*M*,*M*,*P*) chiralities.

### Model Molecules

Three model molecules were chosen to investigate the dependence of RayOA and SR on the degree of chirality in a molecular system. These are hydrogen peroxide (H_2_O_2_), biphenyl, and 1,1′-binaphthalene, which can all have chiral conformations based on the dihedral angle. While chiral conformations of these molecules are not isolable, they allow the degree of chirality in a molecule to be controlled. For H_2_O_2_ and biphenyl, a relaxed scan was performed in steps of 10° from −180° to +180° at the B3PW91/6-311++G­(2d,p) level in the gas phase prior to computing the polarizability tensors at the same level. Because of strong coupling of the scanned 1,1′-binapthalene dihedral angle and others, a rigid scan was instead performed using the low-energy geometry with an internaphthyl dihredral angle of 72°.

#### Comparison of DFT Predictions of SR and RayOA

H_2_O_2_ does not possess a chiral center but can possess *R*_*a*_ or *S*_*a*_ axial chirality due to atropisomerism, i.e., hindered rotation about the O–O bond. While the rotational energy barrier is too low for these atropisomers to be isolable, we can get an estimation of the expected RayOA for two-group type molecules in a chiral orientation as well as dependence of RayOA on the degree of chirality in a system. A plot comparing the SR and RayOA along a relaxed scan of the H–O–O–H dihedral angle is presented in Figure 2. Here, H_2_O_2_ has a *S*_*a*_ configuration when the dihedral angle is between −180° and 0° and has a *R*_*a*_ configuration when between 0° and +180°. Notably, the calculated SR flips the sign for the *S*_*a*_ atropisomer as the dihedral changes between 0 and 180°. This behavior was noted previously,^32^ where prediction of SR was also found to depend on the level of theory used. On the contrary, RayOA retains a consistent, positive sign for the *S*_a_ atropisomer and only switches sign when the *R*_*a*_ atropisomer is formed. The computed SR magnitudes are also quite high, reaching ±170 deg cm^3^/(g dm) at ±120°. Meanwhile, computed magnitudes of RayOA are all below 3.0 × 10^–4^. Overall, these observations suggest that the sign of RayOA is uniquely descriptive of the handedness of the chiral disposition of two O–H groups in H_2_O_2_, while SR is not at the current theoretical level. The reader may refer to the work of Norman and co-workers,^33^ where they indicated that difficulties in the correct prediction of SR in H_2_O_2_ are mainly due to the accidental degeneracy of two lowest excited states. In the case of biphenyl, SR goes through four positive–negative cycles for dihedral angles in the −180° to +180° range (see left panel of Figure 3), but RayOA goes through two positive–negative cycles (see the right panel of Figure 3), as predicted by the two-group model predictions (vide infra).

**Figure 2:**
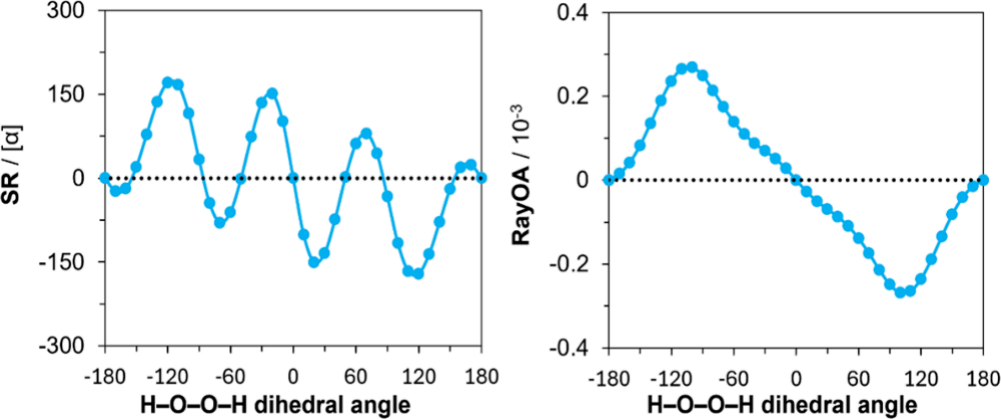
Comparison of SR and RayOA based on the H–O–O–H dihedral angle of hydrogen peroxide at the B3PW91/6-311++G­(2d,p) level in the gas phase at 532 nm. Geometries were obtained from a relaxed scan of the peroxide dihedral angle.

**Figure 3:**
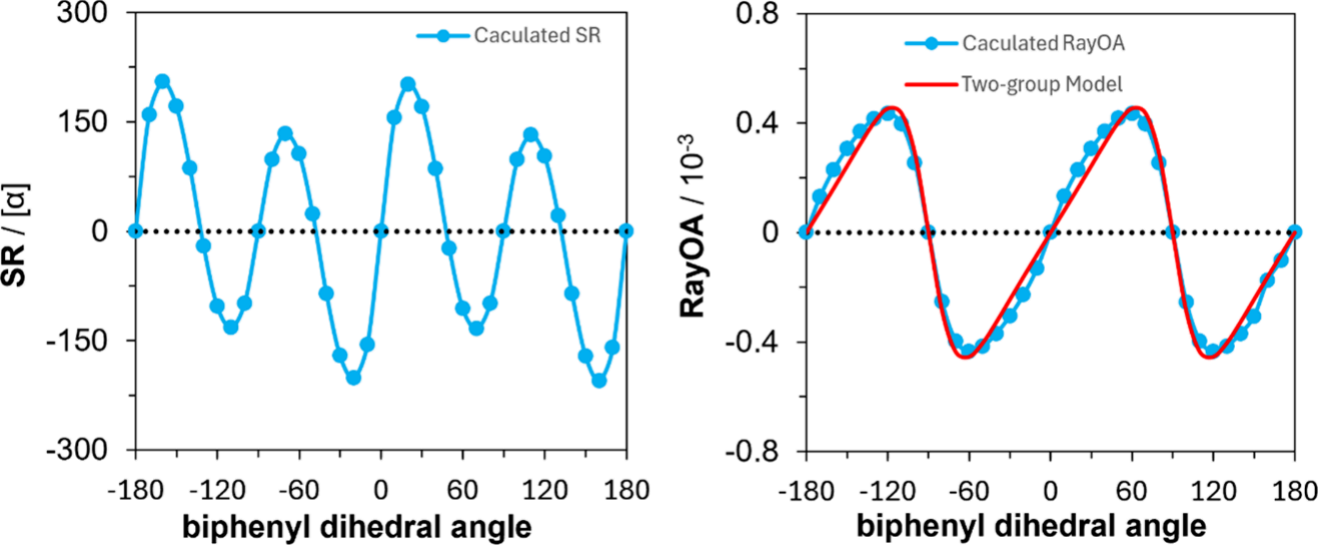
Comparison of SR and RayOA based on the biphenyl dihedral angle in a relaxed scan at the B3PW91/6-311++G­(2d,p) level at 532 nm. In red is the RayOA CID obtained from the two-group model.

#### Evaluation of Simple Models

In 1974, Barron and Buckingham developed a simple two-group model for RayOA and ROA CIDs for the 90° scattering geometry (vide infra), assuming two neutral, optically inactive groups, separated by some distance, *R*, and possessing at least 3-fold or higher axis of rotation.^13^ This model has the advantage of not having to depend on the group polarizabilities or any quantum mechanical information. A generalized two-group model was also presented for RayOA and ROA CID in 1977,^34^ but not pursued here. The atom-dipole interaction model (ADI)^35^ is a classical model that builds the molecular EDED polarizability tensors using spherical atomic polarizabilities and the dipolar interaction between atoms. This ADI model has been adopted for ROA spectroscopy,^36^ but its practical usage suffered from issues associated with obtaining normal coordinate derivatives of the atomic polarizability tensors. These models have garnered little attention since their formulation due to advances in quantum chemical methods. However, if these models can serve as a computationally efficient and reliable means for computing RayOA CIDs, then that would be advantageous for AC determination by RayOA. With this in mind, we now compare quantum chemical predictions with those obtained from the two-group model and the ADI model.

#### Comparison of the Two-Group Model and DFT Predictions of RayOA

For the 90° depolarized ICP scattering geometry, the formula for the RayOA CID in the two-group model is given as

$$\Delta_{z}=\frac{2\pi R\;\sin\left(2\theta \right)}{\lambda [5+3\;\cos\left(2\theta \right)]}$$ 15

where θ is the dihedral angle between the two groups and λ is the wavelength of the incident light. This same equation also applies for a 90° SCP geometry with linear polarization in the scattering plane in accordance with the principle of reciprocity. The validity of this simple model as compared to quantum chemical approaches has yet to be reported, and so, we chose to investigate this in detail using biphenyl as a model molecule. Benzene belongs to the *D*_6*h*_ point group, so phenyl rings can be considered to have 6-fold proper rotation axes. The results from the two-group model and DFT calculations are compared in the right panel of Figure 3 for the dihedral angles between +180° to −180°. An excellent agreement is seen between the two calculations, with a 2θ dependence on the dihedral angle as predicted by eq 15.

It should be noted that when the same comparison is made for H_2_O_2_, B3PW91/6-311++G­(2d,p) level calculations of RayOA for H_2_O_2_ showed θ dependence, and not the 2θ dependence of the two-group model (see the left panel of Figure 4). However, we note that at a lower Hartree–Fock level using the 3-21G basis set, the predicted RayOA for H_2_O_2_ does show 2θ dependence (not shown here).

**Figure 4:**
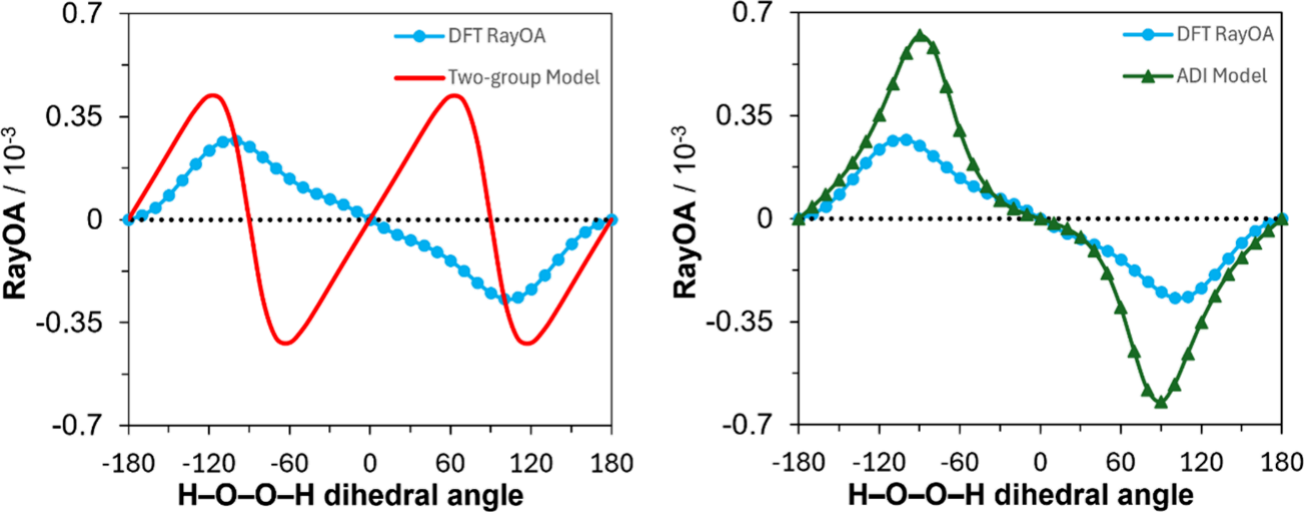
Comparison of RayOA computed for H_2_O_2_ by DFT at the B3PW91/6-311++G­(2d,p) level (blue circles), with that for the two-group model (red), and the atom-dipole interaction model (green triangles). An input wavelength of 532 nm was used.

The SR and RayOA of the third model molecule, binaphthyl, are presented in Figure 5. Since naphthalene belongs to the D_2h_ point group, naphthyl groups do not meet the 3-fold axis requirement of the two-group model. Yet, RayOA values predicted by DFT and the two-group model are in agreement and follow 2θ dependence, as was seen earlier for biphenyl (Figure 3).

**Figure 5:**
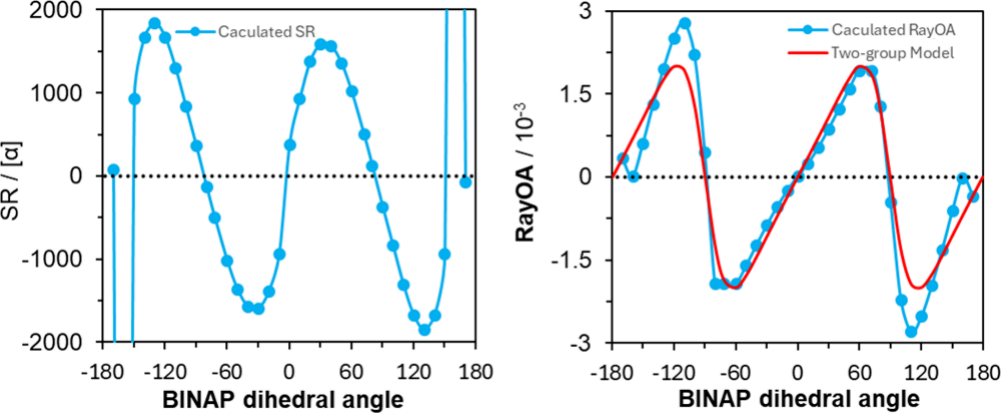
Comparison of RayOA (right panel) computed for binaphthyl at the B3PW91/6-31+G­(2d,p) level (blue circles) with that for the two-group model (red). The left panel shows predicted SR for binaphthyl at the same DFT level. An input wavelength of 532 nm was used. The rigid geometries at ±180° have some H atoms of different naphthyl groups that are too close to each other.

#### Comparison of the ADI Model and DFT Predictions of RayOA

An implementation of the ADI model was coded in Python. This implementation relied on spherical atomic polarizabilities reported by Applequist, which were fitted to available experimental data.^35^ A comparison of RayOA computed with the ADI model, using Applequist’s atomic polarizabilities, is presented in the right panel of Figure 4. It can be seen here that the ADI model captures the dependence of the sign of RayOA CIDs as a function of the H–O–O–H dihedral angle but predicts approximately two times larger magnitudes.

More recently, Litman et al.^37^ reported the atomic polarizabilities fitted to calculations of molecular polarizabilities at Coupled Cluster Singles and Doubles level of theory. The atomic polarizabilities reported by Applequist and Litman et al. vary significantly for the same atom types. If the atomic polarizabilities reported by Litman et al. are used instead,^37^ the dependency on the H–O–O–H dihedral angle resembles that of the two-group model, indicating the sensitivity of the ADI model to the chosen atomic polarizabilities. The availability of a reliable set of spherical atomic polarizabilities appears to be crucial for further use of the ADI model.

### Conformationally Rigid Molecules and Preresonance RayOA

#### α-Pinene

The chiral molecule α-pinene has only one conformer, making it a good example by which to explore the effect of different levels of theory on RayOA and its agreement with experimental data. The wavelength-dependent RayOA calculations for α-pinene were reported before,^22^ but they were not compared with SR. We report that comparison here using several popular functionals for studying the relationship between SR and RayOA and the computed values as a function of wavelength. These functionals are B3LYP,^38,39^ B3PW91,^40^ CAM-B3LYP,^41^ LC-wHPBE,^42^ M06-2X,^43^ and ωB97X-D^44^ utilizing the augmented correlation-consistent Dunning basis set, aug-cc-pVTZ.^45,46^ The SR and RayOA are computed at multiple levels of theory using the same geometry, an optimized geometry of α-pinene obtained from the Supporting Information of previous work,^47^ allowing us to examine the influence of the chosen DFT functional without dependency on differences between structures optimized at different levels of theory. These results are presented in Figure 6. There is remarkable consistency in the predicted RayOA of α-pinene in both the sign and magnitude of RayOA, but only the sign of SR is consistent. This is consistent with a previous report by Zuber at al., who showcased the robustness of RayOA to differing levels of theoretical predictions.^17^

**Figure 6:**
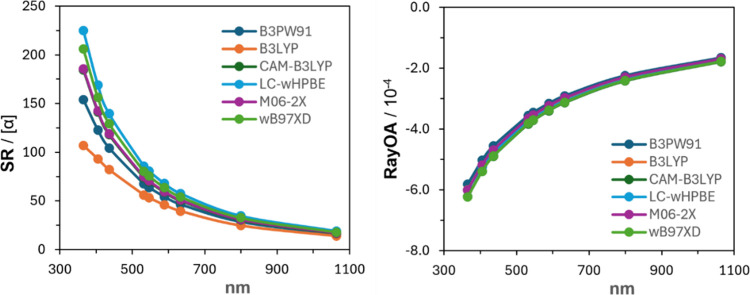
SR (left) and RayOA CIDs (right) for α-pinene at six levels of theory: B3PW91 (dark blue), B3LYP (orange), CAM-B3LYP (dark green), LC-wHPBE (blue), M06-2X (purple), and ωB97X-D (green).

At shorter input wavelengths, the magnitude of SR varies significantly at the DFT functionals used despite RayOA not varying much with the level of theory used. One would expect the SR to change significantly when the wavelength of the SR measurement approaches an electronic transition wavelength. Such effects become dominant for helicenes, as discussed below.

#### Helicenes

Based on the model calculations of SR and RayOA for [6]­helicene, Barron predicted that a molecule with large magnitude SR need not also have a large magnitude RayOA.^14,15^ To gain insight into how RayOA and SR change with wavelength, we set out to investigate the wavelength-dependent RayOA and SR for three helicenes, namely, [5]-, [6]-, and [7]­helicene. These three helicenes can also provide insight into the influence of helicene size on the magnitudes of RayOA and SR.

Optimized geometries of [5]-, [6]-, and [7]­helicene were obtained from the OR45 benchmark,^47^ all with *M* helicity, which were previously optimized at the B3LYP/6-311G­(d,p) level of theory. Polarizability tensors were computed in the gas-phase at the CAM-B3LYP/aug-cc-pVDZ level.

The SR and RayOA curves of (*M*)-[5]-, -[6]-, and -[7]­helicene are presented in Figure 7. Three conclusions coming out of these data are as follows. (1) Both SR and RayOA show negative curves for (*M*)-helicenes. (2) The magnitudes of both SR and RayOA increase with helicene size. (3) At shorter wavelengths, the magnitudes of both SR and RayOA increase significantly.

**Figure 7:**
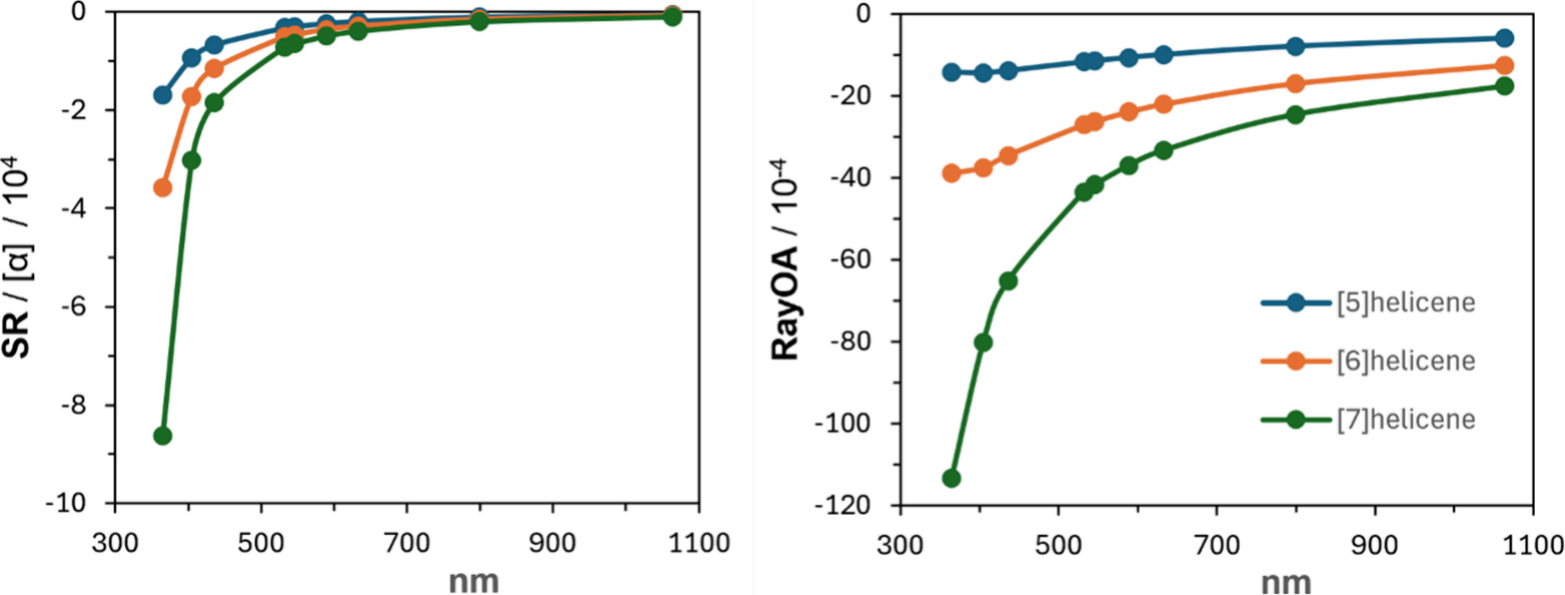
SR and RayOA curves for (*M*)-[5]-, -[6]-, and -[7]­helicene at the CAM-B3LYP/aug-cc-pVDZ//B3LYP/6-311G­(d,p) level.

The sharp increase in SR magnitude with decreasing wavelength can be easily understood in terms of its dependence on ECD bands, through the Kramers–Kronig transform.^48^ Additionally, SR is related to the trace of the ω^–1^ *G*_αβ_^′^ tensor, which in turn depends on the rotational strengths of electronic transitions. The wavelengths of the first 5 electronic transitions and their rotational and dipole strengths of helicenes are summarized in Table 1. The longest wavelength ECD band, with strong negative intensity, in [5]-, [6]-, and [7]­helicene has peak positions at 291, 307, and 330 nm, respectively. For all three helicenes, as the longest wavelength strong negative ECD band gets closer to the input wavelength of SR calculation, the magnitude of SR increases. The magnitude of SR at 365 nm is much higher for [7]­helicene because its strong ECD band at 330 nm is closer to the SR calculation at 365 nm.

**Table 1:** Wavelengths (nm), Rotational (10^–40^ erg-esu-cm/Gauss), and Dipole Strengths (a.u.) for the First Five Electronic Transitions in Helicenes

(*M*)-[5]helicene			(*M*)-[6]helicene			(*M*)-[7]helicene		
wavelength	rotational strength	dipole strength	wavelength	rotational strength	dipole strength	wavelength	rotational strength	dipole strength
337	2.36	0.01	350	0.56	0.03	364	0.09	0.0
316	2.87	0.06	330	1.18	0.01	343	–73.5	0.09
291	–499	5.35	307	–842	4.4	330	–954	2.23
280	–7.34	0.01	296	23.7	0.11	314	–5.24	0.21
277	103	0.64	283	–30.6	0.10	303	39.8	0.16

The sharp increase in magnitude of RayOA with decreasing wavelength can be understood from eqs 4–6, respectively, for α_αβ_, ω^–1^ *G*_αβ_^′^, and *A*_αβγ_ tensors, where the difference ω_*mn*_^2^ – ω^2^ in the denominator decreases when ω approaches ω_mn_. It should be noted that these equations suggest singularity under resonance condition (i.e., at ω_*mn*_ = ω). This singularity is avoided by including line widths in the denominators (see Section 2.6.3 of previous work^8^), and these line widths serve as damping factors. RayOA calculations in the resonance region using dynamic polarizability tensors obtained with linear response theory^49^ by incorporating the damping factors are being evaluated and will be reported at a later date.

An important point that can be noted from this wavelength-dependent investigation on helicenes is that larger magnitude RayOA may be realized when the measurements are made at a wavelength closer to electronic transitions.

### Influence of Conformational Flexibility

#### (1*S*,2*R*,7*S*,8*S*)-(−)-α-Longipinene

For (1*S*,2*R*,7*S*,8*S*)-α-longipinene, a systematic conformational search was performed with the Conflex program using the MMFF94s force field and a search limit of 20 kcal/mol. This process located four conformations that could be divided into two sets of conformations based on the orientation of the seven-membered ring: two low-energy chair conformations (**C1** and **C2**) and two higher-energy (∼8.5 kcal/mol) boat conformations (**C3** and **C4**). All four conformations were initially optimized at the B3PW91/6-31+G­(2d,p) level and then at the B3PW91/6-311++G­(2d,p) level in the gas phase.

The four conformations of (1*S*,2*R*,7*S*,8*S*)-α-longipinene all display the same sign of RayOA, varying in magnitude from ∼2–8 × 10^–4^ (see Table 2 and Figure 8). The SR flips sign between each conformer with both **C1** and **C3** possessing negative SR and **C2** and **C4** possessing positive SR. Conformers **C3** and **C4** are significantly higher in energy (∼8.5 kcal/mol), which means that the sign of Boltzmann-averaged SR relies on both accurate computations of the energy difference between **C1** and **C2**, which is quite small at the present levels of theory (∼0.16 kcal/mol), and accurate predictions of SR for each conformer. This makes AC determination by RayOA especially advantageous in this case, as the sign of predicted RayOA does not depend on these factors.

**Figure 8:**
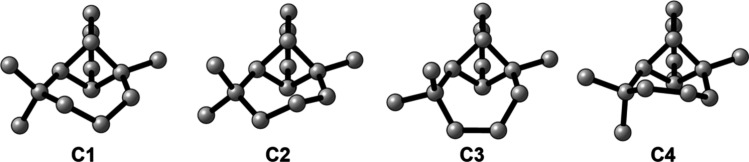
Four conformations of (1*S*,2*R*,7*S*,8*S*)-α-longipinene optimized at the B3LYP/6-311++G­(2d,p) level in the gas phase. Hydrogens have been omitted for the sake of clarity.

**Table 2:** SR and RayOA Values for the Four Conformations of (1*S*,2*R*,7*S*,8*S*)-α-Longipinene with the B3PW91 and CAM-B3LYP Functionals and the 6-311++G­(2d,p) Basis Set

	SR		RayOA (×10^–4^)	
conformer	B3PW91	CAM-B3LYP	B3PW91	CAM-B3LYP
**C1**	–33.59	–51.93	–1.89	–2.07
**C2**	+33.48	+27.42	–7.87	–8.54
**C3**	–49.11	–56.21	–3.86	–3.99
**C4**	+22.55	+5.64	–5.86	–6.46

A common issue with AC determination of flexible molecules by chiroptical spectroscopic methods is accurately correlating the experimental data with ensemble-averaged properties. In the case of large, flexible molecules, this is especially challenging as sacrifices must be made to make costly computations more tractable, often at the expense of reliable computation of molecular properties. The initial calculations of the conformers of α-longipinene, which has limited conformational flexibility, suggest that RayOA may be more robust than traditional chiroptical methods in that RayOA is less sensitive to conformational changes. This is consistent with a previous report by Zuber at al., which showcased the robustness of RayOA to small structural changes.^17^

#### (*R*)-Fluoxetine

To investigate the utility of RayOA in the case of flexible molecules, we performed extensive conformational analysis and optimizations of (*R*)-fluoxetine, which had 351 conformations in the initial ensemble. The conformational ensemble of (*R*)-fluoxetine was optimized using the B3PW91 and M06-2X functionals with the 6-31+G­(2d,p) basis set and PCM representing the methanol solvent. Unique conformations were further optimized at the same level of theory with Grimme’s empirical dispersion corrections: B3PW91-D3B­(J)^50^ and M06-2X-D3.^51^ This resulted in 208 and 201 conformers at the M06-2X and M06-2X-D3 levels, respectively, and 234 and 212 conformers at the B3PW91 and B3PW91-D3B­(J) levels. The needed polarizability tensors were computed for all conformers at all four levels of theory with an input wavelength of 532 nm. The lowest energy conformers within 2.0 kcal/mol were then reoptimized with the 6-311++G­(2d,p) basis set, and then, polarizability tensors were computed at the same level. SR and RayOA were Boltzmann-averaged using electronic energies.

The Boltzmann-averaged RayOA for (*R*)-fluoxetine, which are presented in Table 3, are consistent across all four density functionals using two different basis sets, even though the inclusion of empirical dispersion corrections with either B3PW91 or M06-2X considerably changes the distribution of low-energy conformers. Then, it appears that RayOA is not influenced by the inclusion of dispersion corrections, at least not for (*R*)-fluoxetine. On the contrary, the Boltzmann-averaged SR at 532 nm is not consistent between B3PW91 and other three calculations at B3PW91-D3B­(J), M06-2X, and M06-2X-D3 levels.

**Table 3:** Boltzmann-Weighted SR and RayOA Values for the Ensembles of (*R*)-Fluoxetine with the B3PW91 and CAM-B3LYP Functionals at 532 nm^a^

	6-31+G(2d,p)		6-311++G(2d,p)	
level	SR	RayOA (x10^–4^)	SR	RayOA (×10^–4^)
B3PW91	+16.3 ± 3.8	+16.3 ± 1.0	+4.8 ± 10.0	+17.3 ± 2.0
B3PW91-D3B(J)	–66.6 ± 5.5	+16.6 ± 1.0	–80.5 ± 13.8	+17.9 ± 2.0
M06-2X	–74.2 ± 5.3	+18.4 ± 1.0	–79.3 ± 11.8	+18.1 ± 2.0
M06-2X-D3	–76.2 ± 5.4	+17.5 ± 1.0	–81.2 ± 12.2	+18.1 ± 2.0

a Boltzmann weighting was performed using electronic energies.

A closer examination of the RayOA of individual conformers of (*R*)-fluoxetine with the B3PW91 functional reveals that RayOA remains consistent until approximately 5 kcal/mol (see Figure 9), after which RayOA values flip between −2 × 10^–3^ and +2 × 10^–3^. Low-energy conformers do occasionally flip sign but not until 2 kcal/mol and thus do not meaningfully contribute to the Boltzmann-averaged RayOA. Meanwhile, SR values do not display a similar level of consistency across the low-energy conformers. For conformations having higher energies (≥7.5 kcal/mol for SR and ≥5 kcal/mol for RayOA), further analysis is needed to understand consistent looking sets of SR and RayOA values.

**Figure 9:**
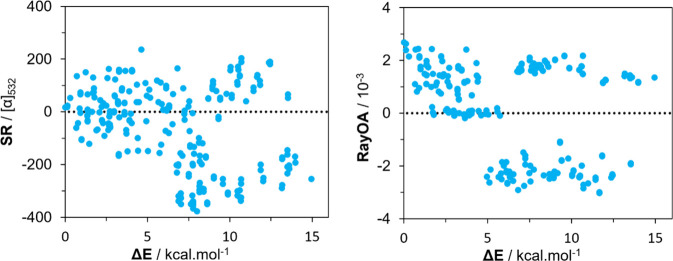
Comparison of SR (left) and RayOA (right) for conformers of (*R*)-fluoxetine at the B3PW91/6-31+G­(2d,p) level.

It is important to note that the calculation to obtain the polarizability tensors needed for the computation of RayOA is relatively simple and that the optimization of the conformational ensemble remains the largest obstacle in obtaining an accurate Boltzmann-averaged RayOA CID. If (1*S*,2*R*,7*S*,8*S*)-α-longipinene and (*R*)-fluoxetine are any indication, it appears that RayOA may be less sensitive to changes in the conformational ensemble, which could be an inherent advantage of AC determination by RayOA.

#### Symmetric Top Molecules

There are only a limited number of molecules belonging to the *D*_3_ point group that have been investigated using chiroptical spectroscopy. Barron and Johnston^16^ used triphenylborane with *D*_3_ symmetry as a model system to predict rotational Raman optical activity and RayOA of symmetric top molecules. Using the group polarizability model, they predicted Δ_*z*_ of −2.61 × 10^–3^ for triphenylborane with left-handed propeller chirality for an input wavelength of 500 nm. Motivated by this encouraging prediction, we sought to verify whether similar magnitude can be predicted by quantum mechanical calculations. We performed quantum mechanical calculations on triphenylborane by optimizing the geometry at the B3LYP/6-31+G­(2d,p) level. The fully optimized geometry has sp^2^ trigonal planar geometry at the boron atom, and phenyl groups were in the propeller orientation, tilted by +34.17°. This right-handed propeller geometry yielded a Δ_*z*_ of +1.75 × 10^–3^ with an input wavelength of 532 nm, which is remarkably close in magnitude to −2.61 × 10^–3^ predicted for the left-handed propeller by Barron and Johnston. This observation gives hope that one may be able to predict RayOA using the approach of Barron and Johnston without having to resort to quantum mechanical calculations in suitable cases.

Motivated by the promising results obtained for the RayOA of triphenylborane, we investigated three additional *D*_3_-symmetric molecules and one *C*_3_-symmetric molecule. These include two chiral explosives, namely, triacetone triperoxide (TATP) and hexamethylene triperoxide diamine (HMTD), and two hydrocarbons, namely, perhydrotriphenylene and triphenylmethane. The first two molecules were previously investigated using theoretical VCD and ROA spectra,^52^ while *D*_3_-symmetric perhydrotriphenylene was investigated by Stephens and co-workers using experimental and theoretical VCD spectra, where a single conformer was found to be dominant at the B3PW91/TZ2P level.^53^ We are not aware of any chiroptical spectroscopic studies on *C*_3_-symmetric triphenylmethane.

Chiral explosives TATP and HMTD possess conformers having *D*_3_ or *C*_2_ symmetry depending on the relative orientation of the dihedral angles.^52^ Although *C*_2_-symmetric molecules generally belong to the category of asymmetric tops, the *C*_2_-symmetric TATP (rotational constants of 0.699, 0.671, and 0.443 GHz) and HMTD (rotational constants of 0.813, 0.755, and 0.731 GHz) are approximate symmetric tops. These molecules serve as good examples to investigate the sensitivity of RayOA to the degree of symmetry in a molecule, as TATP and HMTD can possess either 2-fold or 3-fold symmetry. (*P*,*P*,*P*)-TATP and (*M*,*M*,*M*)-HMTD structures with *D*_3_ symmetry and (*M*,*P*,*P*)-TATP and (*M*,*M*,*P*)-HMTD with *C*_2_ symmetry were used for calculations presented here. The SR and RayOA curves for TATP and HMTD at the CAM-B3LYP/aug-cc-pVDZ level are presented in Figure 10. Here, the SR curves for *C*_2_- and *D*_3_-symmetric structures are very similar in both TATP and HMTD. However, RayOA curves show increased magnitudes for the *D*_3_ structure compared to that with *C*_2_ symmetry. This indicates that RayOA is very sensitive to the degree of chirality (*D*_3_ vs *C*_2_) for a fixed atomic composition. It turned out that SR is relatively insensitive to the *D*_3_–*C*_2_ conformational transition for a fixed atomic composition.

**Figure 10:**
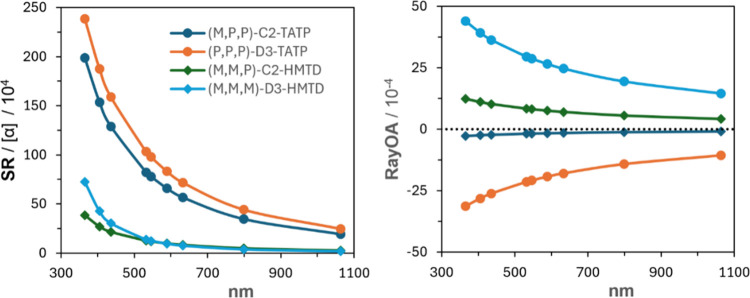
Comparison of SR (left) and RayOA (right) for the *C*_2_- and *D*_3_-symmetric geometries of TATP and HMTD at the CAM-B3LYP/aug-cc-pVDZ level. Geometries were optimized at the B3LYP/6-311++G­(2d,p) level.

Geometries of *D*_3_-symmetric (*S*)-perhydrotriphenylene and *C*_3_-symmetric triphenylmethane with right-handed propeller geometry were optimized at the CAM-B3LYP/aug-cc-pVDZ level, and these optimized geometries were used for RayOA and SR calculations at the same level. In contrast to the above-mentioned triphenylborane and two chiral peroxide explosives, the RayOA magnitude predicted for (*S*)-perhydrotriphenylene is only +4.09 × 10^–5^—a very small value. On the other hand, *C*_3_-symmetric triphenylmethane with right-handed propeller geometry has a RayOA of +4.8 × 10^–3^, which is rather large. This observation suggests that *D*_*3*_ symmetry alone does not guarantee a larger RayOA magnitude but atomic composition plays a definite role. Since greater RayOA results from larger magnitudes of EDMD and EDQD polarizabilities, the predicted low RayOA value for perhydrotriphenylene suggests that molecules containing C and H atoms alone without electron delocalization (such as that present in phenyl rings in triphenylmethane) may not possess larger EDMD and EDQD polarizabilities.

#### Triphenylborane-Ammonia Complexes

Additionally, several propeller chiral molecules were also investigated: triarylborane, (amino)­(triphenyl)­boron, and derivatives of borane ammonia complexes (see Figures 1 and 11). For all of these molecules, optimizations were performed at the B3LYP/6-31+G­(2d,p)/PCM­(CHCl_3_) level, and polarizability tensors were computed at the same level.

**Figure 11:**
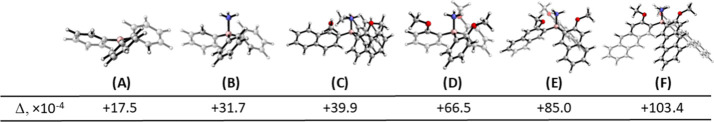
RayOA CIDs of right-handed propeller chiral triarylborane molecules at the B3LYP/6-31+G­(2d,p)/PCM­(CHCl_3_) level at 532 nm. Geometries are optimized at the same level prior to the calculation of polarizability tensors. Molecules shown here are (A) triphenylborane, (B) triphenylborane ammonia, (C) tri-1-methoxynaphthylborane ammonia, (D) trimethoxyphenylborane ammonia, (E) tri-3-methyoxynaphthylborane ammonia, and (F) trimethoxyanthraceneborane ammonia.

Merten and co-workers have reported the synthesis and VCD studies on triphenylborane-ammonia complexes.^54,55^ The optimized geometries of these molecules were obtained from the Supporting Information of their articles. The computed RayOA CIDs at these geometries had magnitudes as large as 1.25 × 10^–2^ (specifically for some conformers from elsewhere,^54^ see Table S1 and Figure S1), which is 2 orders of magnitude larger than a typical RayOA CID at 532 nm (vide supra). These triarylborane ammonia complexes feature propeller chirality due to steric hindrance of the triaryl subunits attached to boron, in addition to stabilization of the chiral propeller conformations due to the four-point interaction of the complexed ammonia molecule. Merten and co-workers investigated derivatives of these complexes in which point chiral groups were introduced either on each of the triaryl subunits^55^ or on the complexed amine.^54^ In addition to these triphenylborane derivatives, we also investigated a tetradecacyclic diborate with 1,1′-binaphthyl “blades” providing the propeller structure^56^ with *M* chirality (see Figure S2). This molecule is predicted to have a RayOA CID of −4.05 × 10^–3^, which is also fairly large.

For understanding the large magnitudes of RayOA in triarylborane ammonia complexes with propeller chirality, it is important to note that right-handed propeller triphenylborane (Figure 11A), with trigonal planar geometry at boron, itself has RayOA CID of +1.75 × 10^–3^. Then, the addition of NH_3_ (see triphenylborane ammonia, Figure 11B) distorts the trigonal planar central atom to a tetrahedral geometry and nearly doubles the RayOA magnitude to +3.17 × 10^–3^ (comparable to that of triphenylmethane mentioned above). Adding a methoxy group at the C2 position adjacent to the boron atom (see trimethoxyphenylborane ammonia, Figure 11D) results in a four-point interaction stabilized complex studied by Merten et al. (molecule **1a** from previous work^55^) and again doubles the RayOA magnitude to +6.65 × 10^–3^. Swapping out the triphenyl subunits with larger aromatic subunits (naphthyl or anthracene) further increases the RayOA magnitudes. With trianthracene subunits (see Figure 11F), the RayOA magnitude increases to +1.03 × 10^–2^. These initial data suggest that triarylborane can serve as a potential scaffold for designing Rayleigh scatterers with large magnitude of RayOA. However, extending the size of the aromatic subunits does not guarantee an increased magnitude of RayOA. If the aryl subunits are extended at the C3 and C4 positions (see tri-1-methoxynaphthylborane ammonia, Figure 11C), instead of the C4 and C5 positions (see Figure 11E), then the RayOA decreases relative to the trimethoxyphenylborane ammonia complex.

To investigate why lengthening the triaryl units in the triarylborane scaffold significantly increased the magnitude of RayOA, the ω^–1^G’ tensors were visualized as tensor surface representations (see Figure 12). These data indicate that the increased RayOA magnitudes arise from increasing the magnitudes of the positive and negative surface contributions, which increases the overall anisotropy.

**Figure 12:**
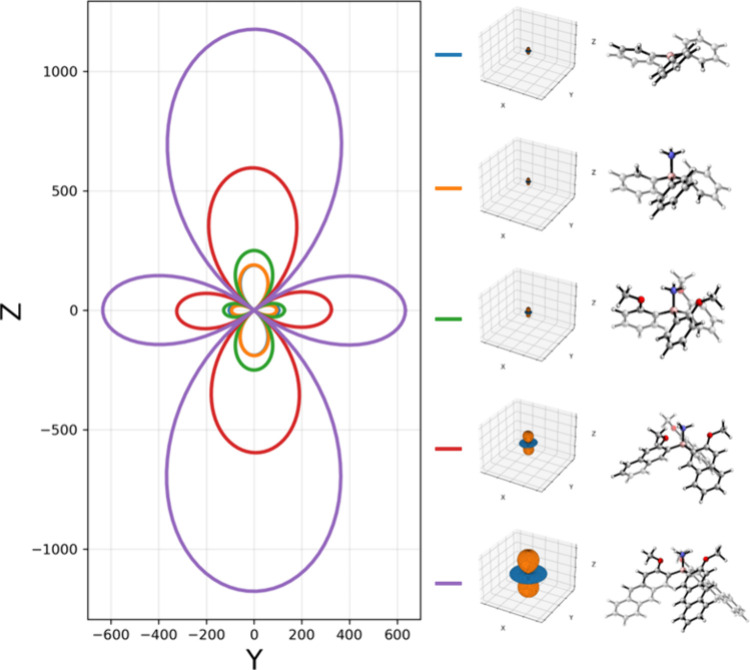
EDMD tensor surface representations for the right-handed propeller triarylborane molecules at the B3LYP/6-31+G­(2d,p)/PCM­(CHCl_3_) level using an input wavelength of 532 nm. Presented is an overlay of the cross sections of all of the visualized tensors. The trace for triphenylborane is hidden behind that for triphenylborane ammonia.

#### Solute–Solvent Clusters: Incremental Solvent Addition to Dimethytartrate and Tartaric Acid^57^

One of the obstacles in the routine assignment of AC by chiroptical spectroscopy^58–60^ is the presence of strong intermolecular interactions, which can lead to solute–solvent clusters and molecular aggregates. The presence of these intermolecular interactions forms long-lived, spectroscopically observable clusters or aggregates, which must be incorporated into the computational treatment of the molecule in question. This computational treatment is nontrivial, as generation of aggregate structures and subsequent exploration of the potential energy surface are difficult and thus can preclude routine AC assignment.^61^ In certain cases, such as methanol, chiroptical spectra can be interpreted without explicit modeling of solvent molecules, but this is difficult to know a priori.^62^ The sensitivity of chiroptical spectroscopies such as VCD and ROA to these intermolecular interactions can be seen as a disadvantage in this case. This raises the question whether RayOA is also sensitive to these interactions.

To further investigate the utility of RayOA for AC assignment, we need to properly investigate a system with strong intermolecular interactions. For this purpose, we chose dimethyl tartrate (DMT) and tartaric acid (TA), which was studied previously for VCD in DMSO solvent.^57^ The modeling of solute–solvent intermolecular interactions was found to be crucial to interpreting the VCD spectra in DMSO. Conformations for solute–solvent clusters of DMT and TA were taken from that work. DMT solute–solvent clusters ranged from 0 to 2 DMSO molecules and 0 to 4 DMSO molecules for TA. Polarizability tensors were computed for all previously optimized geometries at the ωB97X-D/6-311++G­(2d,2p)/PCM­(DMSO) level. SR and RayOA were Boltzmann-averaged using electronic and zero-point energies (ZPEs). The Boltzmann-averaged SR and RayOA of DMT and TA with an input wavelength of 532 nm are reported in Table 4. Due to potential issues with accurate entropic contributions to low-frequency vibrational modes of solute–solvent complexes, electronic energies including ZPEs are used for Boltzmann weighting instead of Gibb’s free energies.

**Table 4:** SR and RayOA Values for (*S*,*S*)-Dimethyl Tartrate (DMT) and (*S*,*S*)-Tartaric Acid (TA) with 0–2 and 0–4 Explicit DMSO Molecules^a^

	electronic energies		ZPEs	
molecules	SR	RayOA (×10^–4^)	SR	RayOA (×10^–4^)
DMT-PCM	+20.07 ± 16.48	+2.72 ± 0.54	+39.71 ± 14.88	+2.69 ± 0.39
DMT:1DMSO	+53.40 ± 53.40	+5.93 ± 0.92	+63.71 ± 43.87	+6.03 ± 0.89
DMT:2DMSO	+70.19 ± 28.13	+3.67 ± 2.41	+54.05 ± 26.08	+2.15 ± 2.79
TA-PCM	+30.97 ± 7.10	+1.38 ± 0.69	+28.13 ± 9.22	+3.68 ± 0.92
TA:1DMSO	+16.15 ± 15.95	–6.81 ± 0.90	–10.53 ± 8.38	+1.31 ± 0.51
TA:2DMSO	–17.20 ± 14.17	+5.52 ± 0.42	+0.26 ± 16.95	+5.43 ± 0.47
TA:3DMSO	–58.76 ± 16.83	+4.52 ± 0.58	–55.23 ± 16.97	+4.61 ± 0.56
TA:4DMSO	+62.96 ± 18.16	+4.64 ± 0.99	+57.56 ± 17.50	+4.85 ± 0.93

a An input wavelength of 532 nm was used.

The conformational ensembles of DMT range from no DMSO molecules to two DMSO molecules. All three ensembles have PCM treatment of long-range solvent interactions. For DMT, there is little variation in the predicted RayOA if either electronic energies alone or electronic energies including ZPEs are used to the Boltzmann average. However, the magnitudes of SR are dependent on the type of Boltzmann averaging and strongly dependent on the number of explicit solvent molecules included in the calculation. The inclusion of two solvent molecules, which was necessary for complete interpretation of the VCD spectra in DMSO,^57^ more than triples the SR (when electronic energies are used) as compared to DMT with only PCM treatment. In the case of RayOA, magnitude remained about the same for the inclusion of 0 and 2 solvent molecules. With the inclusion of one DMSO molecule as compared to structures with PCM treatment alone, the RayOA magnitude has increased. While the sign of Boltzmann-averaged RayOA is consistent across all three DMT ensembles, there is a larger uncertainty for the DMT:2DMSO ensemble. This is surprising because these geometries were able to successfully interpret the VCD spectra in DMSO-*d*_6_,^57^ indicating that the solute–solvent dynamics are accurately represented.

The conformational ensembles for TA have 0–4 DMSO molecules, with all ensembles having PCM treatment of long-range solute–solvent interactions. For ensemble-averaged RayOA with 0–1 DMSO molecules, there is some variability in the predicted RayOA with inconsistency in the predicted RayOA of TA:1DMSO if one uses electronic energies alone or electronic energies including ZPEs for Boltzmann averaging. With ZPE based Boltzmann averaging, RayOA CID has consistent sign from including no explicit solvent molecule to including 4 solvent molecules. Additionally, the uncertainties for SR are quite high, while the uncertainties for RayOA are an order of magnitude smaller.

#### Zero-Point Contributions

Another point of consideration is the possible influence of zero-point vibrational contributions, first studied by Ruud et al.,^63^ to the SR of chiral molecules. This phenomenon has been further explored recently by several researchers, also focusing on the effect of deuterium substitution on SR.^64,65^ Zero-point vibrational contributions have been demonstrated to be important in the case of small or low SR values^66,67^ and in the case of solvation and SR sign change.^68,69^ Whether zero-point vibrational contributions are important in the case of RayOA, especially in cases where it is significant for SR, remains to be investigated.

## Conclusions

This work builds upon recent breakthroughs in the experimental observation of RayOA for chiral molecules and reports on the potential utility of RayOA for the determination of absolute configurations. The conclusions emerging from this investigation can be summarized as follows:

(1) The RayOA predictions obtained from the two-group model are in agreement with those obtained from quantum mechanical predictions for two model molecules, biphenyl and binaphthyl. (2) The applicability of the ADI model for RayOA predictions needs the development of reliable values for spherical atomic polarizabilities. (3) For conformationally rigid molecules, like α-pinene, RayOA is fairly insensitive to the level of theory used for its predictions. (4) Conformationally rigid helicenes revealed enhanced RayOA magnitudes when the wavelength of measurement approaches that of electronic transitions. This observation opens up a new area, namely, resonance RayOA. (5) For conformationally flexible molecules, (1*S*,2*R*,7*S*,8*S*)-(−)-α-longipinene and (*R*)-fluoxetine, predicted RayOA is found to be relatively insensitive to conformational changes due to their flexibility. (6) RayOA is found to be robust for the dispersion interactions in (*R*)-fluoxetine. This is an important observation that needs further investigations on additional molecules. (7) An opportunity is identified for designing molecules with large magnitudes of RayOA as seen for chiral propellers (boranes and triarylmethane) and chiral peroxide explosives. (8) RayOA appears to be fairly independent of incremental addition of solvent molecules in dimethyl tartrate-dimethyl sulfoxide and tartaric acid-dimethyl sulfoxide solute–solvent clusters.

All these observations suggest that the variables (such as level of theory, conformational flexibility, dispersion interactions, and solute–solvent clusters) that plague other chiroptical methods could be less of a problem for RayOA predictions. Nevertheless, it is important to investigate additional molecules for wider validity of these observations. We hope that the current extensive observations on the potential utility of RayOA as a chiroptical tool can excite the chemical and chiroptical communities.

## Supplementary Material



## Notes

Table S1: Computed RayOA of triphenylborane ammonia complexes from the Supporting Information of ref 54 at the B3LYP/6-31+G­(2d,p)/PCM­(CHCl3) level. Propeller chiralities are indicated by *P* or *M*. Figure S1: Renderings of triphenylborane ammonia complexes from refs 54 and 55. Figure S2: Optimized geometry of a tetradecacyclic diborate with 1,1′-binaphthyl “blades” providing propeller structure (ref 56) at the B3LYP/6-31+G­(2d,p) level. Computed RayOA CID at the same level is −4.05 × 10^–3^ (PDF)
