# Supplementary material for: Investigation of the Robustness of Rayleigh Optical Activity for the Assignment of Absolute Configurations of Chiral Molecules
Source: J Phys Chem A. 2026 Feb 25;130(10):2238–51. doi: 10.1021/acs.jpca.5c08390 (PMC12990121; doi:10.1021/acs.jpca.5c08390)
Supplement: Supplementary file 1 [file jp5c08390_si_001.pdf]

Supporting Information:
Investigation of the Robustness of Rayleigh Optical Activity for the Assignment of Absolute Configurations of Chiral Molecules

Andrew R. Puente,[1] Duncan McArthur,[2] and Emmanouil I. Alexakis[2], Lewis E. MacKenzie[3], Robert P. Cameron[2], Laurence D. Barron[4], and Prasad L. Polavarapu[1*]

Summary of Supplementary Information

Table S1: Computed RayOA of triphenylborane ammonia complexes from Supp. Info. of Ref. 54 at the B3LYP/6-31+G(2d,p)/PCM($CHCl_3$) level. Propeller chiralities are indicated by P or M.

Figure S1: Renderings of triphenylborane ammonia complexes from Refs. 54 and 55.

Figure S2: Optimized geometry of a tetradecacyclic diborate with 1,1'-binaphthyl "blades" providing propeller structure (Ref. 56) at the B3LYP/6-31+G(2d,p) level. Computed RayOA CID at the same level is -4.05×10^{-3}.

Table S1. Computed RayOA of triphenylborane ammonia complexes from Supporting Information of Ref. 54 at the B3LYP/6-31+G(2d,p)/PCM($CHCl_3$) level. Propeller chiralities are indicated by *P* or *M*.

Conformer	Chirality	RayOA		Conformer	Chirality	RayOA
1b-C1	*P*	6.60E-03		1c-C1	*P*	6.61E-03
1b-C2	*M*	-9.55E-03		1c-C2	*M*	-1.25E-02
1b-C3	*P*	5.74E-03		1c-C3	*M*	-9.11E-03
1b-C4	*P*	5.27E-03		1c-C4	*M*	-9.76E-03
1b-C5	*P*	5.24E-03		1c-C5	*M*	-8.35E-03
1b-C6	*M*	-7.42E-03		1c-C6	*M*	-1.06E-02
1b-C7	*M*	-7.03E-03		1c-C7	*M*	-1.06E-02
1b-C8	*M*	-7.72E-03		1c-C8	*M*	-7.28E-03
1b-C9	*M*	-6.62E-03		1c-C9	*M*	-7.45E-03
1b-C10	*P*	5.82E-03		1c-C10	*P*	7.99E-03
2b-C1	*P*	2.89E-03		2c-C1	*P*	8.44E-03
2b-C2	*M*	-3.10E-03		2c-C2	*M*	-5.66E-03

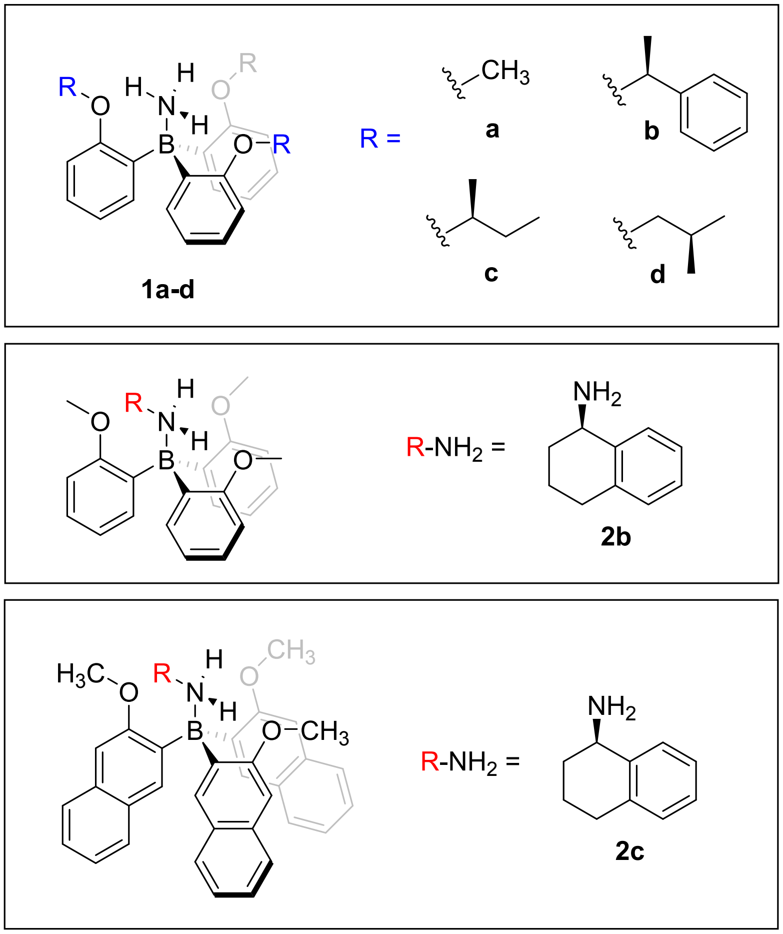

Figure S1. Renderings of triphenylborane ammonia complexes from Ref. 54 and 55.

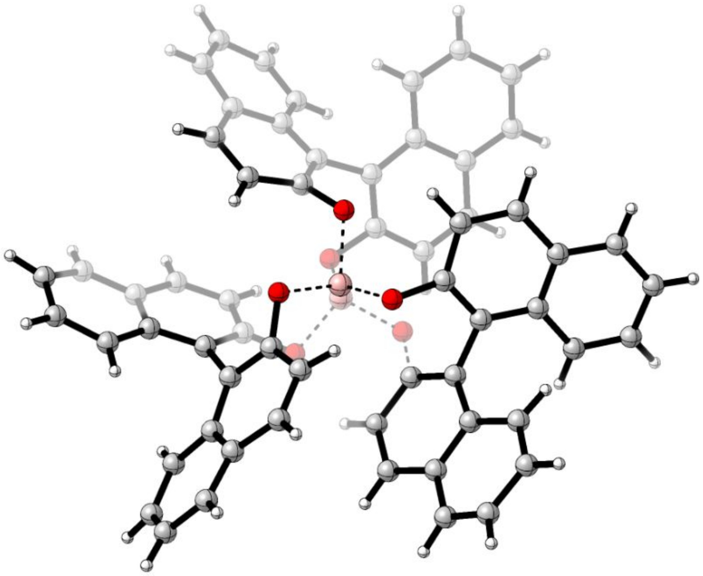

Figure S2. Optimized geometry of a tetradecacyclic diborate with 1,1'-binaphthyl "blades" providing propeller structure (Ref. 56) at the B3LYP/6-31+G(2d,p) level. Computed RayOA CID for this molecule at the same level is -4.05×10^{-3}.
